# Factors influencing the biodiversity of three microbial groups within and among islands of the Baltic Sea

**DOI:** 10.1093/femsec/fiab049

**Published:** 2021-03-22

**Authors:** Anette Teittinen, Leena Virta, Mingjia Li, Jianjun Wang

**Affiliations:** Department of Geosciences and Geography, P.O. Box 64, FI-00014 University of Helsinki, Finland; Department of Geosciences and Geography, P.O. Box 64, FI-00014 University of Helsinki, Finland; Tvärminne Zoological Station, University of Helsinki, J.A. Palménin tie 260, 10900 Hanko, Finland; State Key Laboratory of Lake Science and Environment, Nanjing Institute of Geography and Limnology, Chinese Academy of Sciences, 73, East Beijing Road, Nanjing, Jiangsu 210008, China; University of Chinese Academy of Sciences, 19, Yuquan Road, Shijingshan District, Beijing 100049, China; State Key Laboratory of Lake Science and Environment, Nanjing Institute of Geography and Limnology, Chinese Academy of Sciences, 73, East Beijing Road, Nanjing, Jiangsu 210008, China; University of Chinese Academy of Sciences, 19, Yuquan Road, Shijingshan District, Beijing 100049, China

**Keywords:** bacteria, beta diversity, cyanobacteria, diatoms, islands, species richness

## Abstract

Islands provide ideal model systems to examine the factors influencing biodiversity, yet knowledge of microbial biodiversity on islands remains scarce. We collected a dataset from 101 rock pools along a freshwater to brackish water transition on islands of the Baltic Sea and investigated the patterns and drivers of community composition and species richness of diatoms, cyanobacteria and non-cyanobacteria bacteria among islands. We also examined whether environmental heterogeneity increased beta diversity and species richness within islands. Among islands, the patterns in community composition were concordant among the microbial groups, with distinct changes along the freshwater–brackish gradient. The patterns in species richness were context-dependent for each microbial group. In general, richness patterns were most strongly associated with nutrient concentrations or the distances to potential sources of immigrants, whereas no positive relationships between ecosystem size and richness were found. Within islands, environmental heterogeneity was positively correlated with the beta diversity of each microbial group, but not species richness. Our findings provide novel insights into the factors influencing microbial biodiversity. The results suggest that island microbial biodiversity patterns are influenced by species sorting and dispersal-related mechanisms and highlight the importance of environmental heterogeneity for beta diversity.

## Teaser

This study examines microbial biodiversity patterns and their determinants in rock pools within and among islands of the Baltic Sea.

## INTRODUCTION

Islands have long served as ideal model systems to examine the mechanisms underlying biodiversity patterns and island studies have contributed enormously to ecological and evolutionary theories (MacArthur and Wilson 1967; Warren *et al*. 2015). Islands are also valuable for the variety of life on Earth as they host high numbers of endemic species and collectively contribute to global biodiversity disproportionately to their area (Whittaker and Fernández-Palacios 2007). The majority of insights into island biodiversity stem from studies on larger organisms, however, knowledge of the patterns and drivers of microbial communities on islands remains limited (but see Davison *et al*. 2018 and Wang *et al*. 2019 for soil microbes). Given that microorganisms have indispensable roles in ecosystem functioning and biogeochemical cycles (Falkowski, Fenchel and Delong 2008; Hope, Paterson and Thrush 2020), increased understanding of the factors shaping their biodiversity is essential.

One of the most renowned theories to explain variation in species diversity is MacArthur and Wilson's (1967) theory of island biogeography. The theory suggests that the number of species on an island is determined by island size and isolation, which in turn regulate species immigration and extinction rates. Species richness is predicted to increase with island area and decrease with isolation from potential sources of immigrants (MacArthur and Wilson 1967). Building partly on the foundations laid by the theory of island biogeography, metacommunity ecology has since then emerged to explain the assembly of biological communities by the interplay of local and regional factors (Leibold *et al*. 2004; Leibold and Chase 2018). Recently, investigating whether microorganisms exhibit biodiversity patterns resembling those often observed for macroorganisms and the degree to which local environmental conditions and regional processes explain microbial communities has increased. Several studies have reported that the key process affecting microbial communities may be species sorting (e.g. Beisner *et al*. 2006; Van der Gucht *et al*. 2007; Souffreau *et al*. 2015; Rodríguez-Alcalá *et al*. 2020), which emphasizes the role of local environmental factors in controlling communities and assumes sufficient dispersal facilitating species occurrence in environmentally suitable sites (Leibold *et al*. 2004). Others have, however, found spatially structured patterns in microbial communities that cannot be entirely explained by contemporary environmental variability, resulting thus also from historical and dispersal-related processes (e.g. Langenheder and Ragnarsson 2007; Vyverman *et al*. 2007; Soininen *et al*. 2016). Despite increased efforts to disentangle the processes structuring microbial communities, no consensus has been reached as to when and why certain processes prevail over others.

To examine the roles of environmental and spatial factors affecting communities, rock pools provide suitable venues as they may be envisioned as islands in a terrestrial landscape or as habitat patches in a metacommunity (Ebert, Hottinger and Pajunen 2001; Langenheder *et al*. 2012). They often occur in hierarchically structured clusters, and are small, structurally simple ecosystems with clear boundaries, facilitating relatively straightforward definitions of local communities (Srivastava *et al*. 2004). Rock pools are small, water-filled bedrock depressions occurring in landscapes where environmental conditions, such as wind and wave action, prevent the accumulation of detritus and vegetation on the surface of bedrock (Pajunen and Pajunen 2007). They are typically characterized by high environmental variability; for instance, rock pool salinity and nutrient concentrations may differ considerably even within a single island (Pajunen and Pajunen 2003). Community assembly in rock pools may be affected not only by such environmental heterogeneity, but also by spatial factors and species dispersal connecting rock pools to each other and the surrounding region (Hanski and Ranta 1983; Langenheder and Ragnarsson 2007; Aarnio, Teittinen and Soininen 2019). Earlier research on rock pool biota has mostly focused on larger organisms, such as aquatic invertebrates (Hanski and Ranta 1983; Pajunen and Pajunen 2003; Vanschoenwinkel, Buschke and Brendonck 2013), but to our knowledge, no study has examined rock pool microbial communities using multiple taxonomic groups.

Here, our aim is to unravel the key factors affecting the biodiversity of three microbial groups using a dataset collected from 101 rock pools on 10 islands on the coast of the Baltic Sea. The studied microbial groups comprise diatoms (i.e. unicellular algae), cyanobacteria and non-cyanobacteria bacteria (i.e. bacteria other than cyanobacteria), groups of which have vital roles in multiple ecosystem functions. We focus on these three taxonomic groups because they have broadly different functional roles in ecosystems as diatoms and cyanobacteria represent primary producers while non-cyanobacteria mostly represent decomposers. The specific aims of this study are (i) to decipher the roles of environmental and spatial factors in shaping community compositions among islands, (ii) to examine whether species richness is primarily associated with water chemistry or pool size and distances to potential sources of immigrants as predicted by the theory of island biogeography (MacArthur and Wilson 1967), and (iii) to test whether environmental heterogeneity increases beta diversity and species richness within islands (Stein, Gerstner and Kreft 2014).

## MATERIALS AND METHODS

### Study area and rock pools

**Figure 1.**
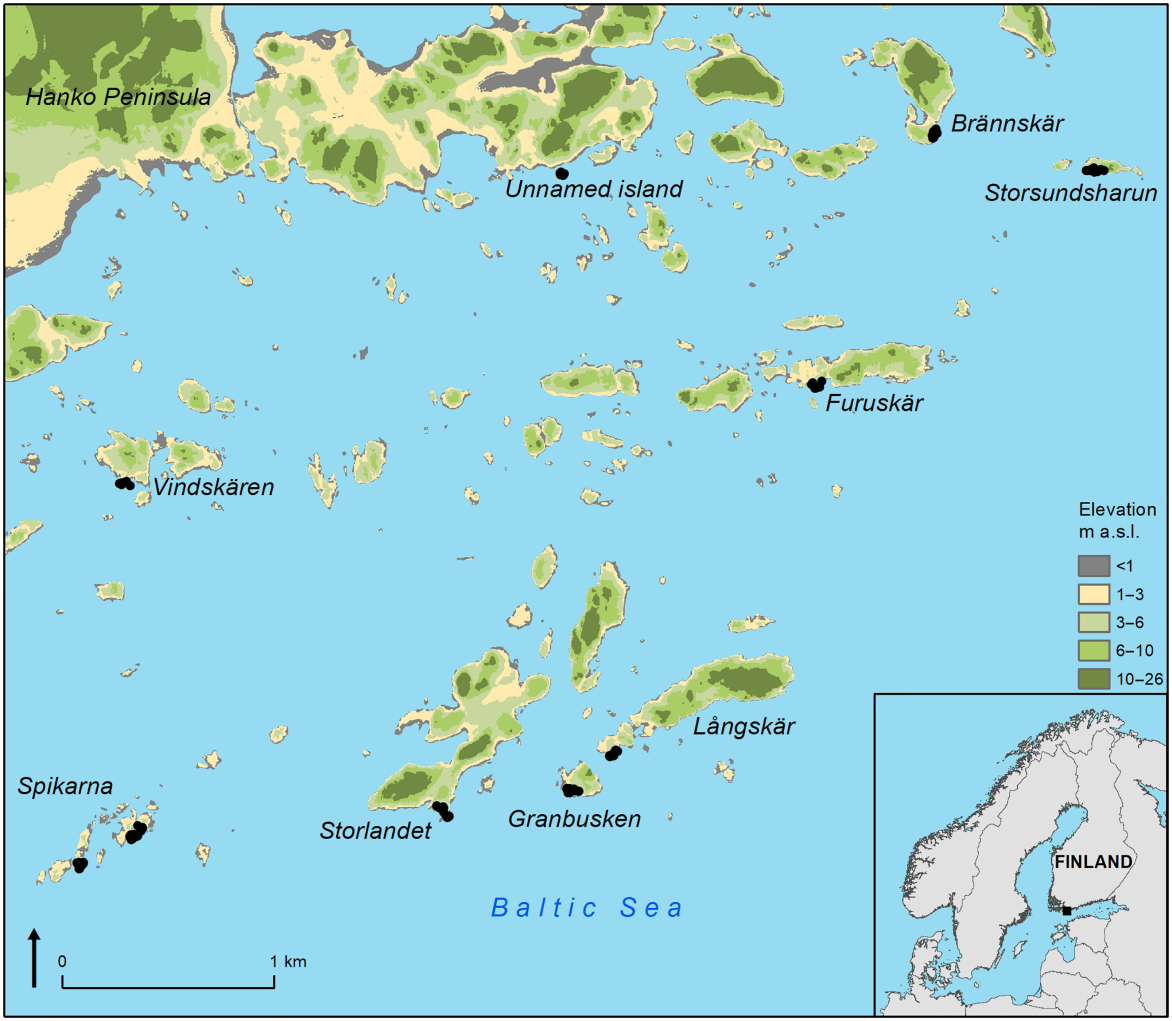
Map of the study area, showing the locations of the 10 sampled islands and 101 rock pools on the coast of the Baltic Sea, southern Finland (59°48′–59°51′N, 23°12′–23°18′E). Contains data from the National Land Survey of Finland, Elevation model 11/2019; Finnish Environment Institute, Water formations 2016; GADM.

The study area is located on the coast of the Baltic Sea, in southern Finland (59°48′ to 59°51′N, 23°12′ to 23°18′E) (Fig. 1). We sampled 101 rock pools located on 10 islands differing in size and distance to the mainland of Finland, comprising six larger, wooded islands and four smaller woodless skerries. On each island, we aimed at sampling a mixture of different habitats, ranging from freshwater pools in the more sheltered interior parts of the islands to more exposed brackish pools closer to seashore. Sampling was carried out in June 2018 within 2 weeks to reduce the effects of temporal variation on pool microbial communities and environmental conditions. At the time of the sampling, all pools were unshaded and hydrologically independent, that is, not connected by watercourses to each other or the sea. All pools were located at low elevations (<15 m a.s.l.) and embedded on the exposed surface of the bedrock, consisting mainly of quartz diorite and granodiorite.

Owing to the small size of rock pools, their physicochemical properties are tightly linked to the surrounding environment and climatic conditions. They experience high climatic variation as the regional mean monthly temperature ranges from −4.0°C in February to +17.2°C in July (normal period 1981–2010; Pirinen *et al*. 2012). The most common wind direction in the region is southwest. Therefore, the samples were collected mainly from the southwestern side of islands where the zone of exposed bedrock surface is typically widest.

### Field sampling and laboratory methods

Biofilm was collected by scraping the submerged surface of the bedrock with a sterilized sponge. From each rock pool, 10 subsamples of 100 cm^2^ were collected from different sides of the pool. The subsamples were combined into a composite sample and subsequently divided into one sample container for diatoms and one sample container for bacteria (including cyanobacteria and non-cyanobacteria). The diatom samples were preserved with ethanol and stored in the cold (+4°C) and dark until laboratory analyses. The bacteria samples were stored frozen (−20°C).

Water pH, conductivity and temperature were measured in situ using a Hach HQ40d multimeter (Hach, Loveland, CO, USA). Water samples were collected and analyzed in the laboratory for total nitrogen (TN) according to standard SFS-EN ISO 11 905–1 and for total phosphorus (TP) according to SFS-EN ISO 6878. The dimensions of each pool were recorded by measuring maximal length, maximal width (perpendicular to length) and maximal depth. Based on these measurements, pool surface area (length × width) was calculated and pool volume estimated as an inverted pyramid (length × width × depth /3) (Ebert, Hottinger and Pajunen 2001). For each pool, the shortest distance to the seashore and neighboring pools was measured in situ, and isolation was calculated as the pool's mean distance to five closest pools (Pajunen and Pajunen 2007). Elevation was recorded with a Global positioning system (GPS) device. The locations of the rock pools were recorded using a GPS and marked on printed orthophotos in the field. Due to the close proximity of the pools to each other within islands, exact coordinates were extracted later using digital maps and orthophotos, as was also the shortest distance to the mainland of Finland (i.e. the hypothesized source of freshwater species) from each pool and the size of each island.

### Diatom analysis

In the laboratory, diatom samples were cleaned of organic material using wet combustion with hydrogen peroxide (30% H_2_O_2_) and mounted on slides with Naphrax. Using a phase contrast light microscope (Olympus BX40, Melville, NY, USA; magnification 1000 ×), 500 frustules per sample were identified to the lowest possible taxonomic level (typically species level) according to Krammer and Lange-Bertalot (1986, 1988, 1991a,b), Snoeijs (1993), Snoeijs and Vilbaste (1994), Snoeijs and Potapova (1995), Snoeijs and Kasperovicienè (1996) and Cantonati, Kelly and Lange-Bertalot (2017). Species counts were transformed into relative abundances and local species richness was calculated as the sum of all observed species in each pool.

### Bacterial analyses

For bacterial communities, we followed the same procedures as described in Wang *et al*. (2017). Biofilm DNA was extracted using a DNeasy PowerSoil DNA Isolation Kit (QIAGEN, Germany) and the quality was assessed based on ratios of absorbance at 260/280  nm around 1.8 and 260/230 nm >1.7) detected by a NanoDrop ND-1000 spectrophotometer (NanoDrop Technologies, Wilmington​, DE, USA). Bacterial 16S rRNA genes were amplified by bacterial universal primers (515F, 5′-GTGCCAGCMGCCGCGGTAA-3′ and 806R, 5′-GGACTACHVGGGTWTCTAAT-3′) targeting the V4 region in triplicate. Positive PCR products were confirmed by agarose gel electrophoresis, and the PCR products from triplicate reactions were combined and quantified with PicoGreen (Eugene, OR, USA). PCR products from samples to be sequenced in the same MiSeq run were pooled at equal molality to maximize the even-sequencing efforts for all samples. The mixtures were purified with a QIAquick Gel Extraction Kit (QIAGEN, Germantown, MD, USA) and requantified with PicoGreen. Sequencing libraries were prepared according to the MiSeq Reagent Kit Preparation Guide (Illumina, San Diego, CA, USA) and sequenced (Magigene, China).

The raw reads of 16S were merged as single sequence by using the FLASH program (Magoč and Salzberg 2011), with forward and reverse reads overlapped at least 10 bp. FastQC v0.11.8 was used to check the Phred quality of the sequence and the frequency of potentially contaminated adapters (Andrews 2010). The sequences were then trimmed using the paired end mode of Trimmomatic v0.39 with the average Phred quality within a 4-bp sliding window lower than 25 and the remaining sequences shorter than 250 bp were also discarded (Bolger, Lohse and Usadel 2014). The rest of the sequences were analyzed using Quantitative Insights Into Microbial Ecology (QIIME) pipeline version 1.9.1 (Caporaso *et al*. 2010). Sequences longer than 200 bp were denoised with the Denoiser algorithm (Reeder and Knight 2010) and clustered into operational taxonomic units (OTUs) at 97% similarity level with the seed-based UCLUST algorithm (Edgar 2010). The taxonomic identity of each representative sequence was determined using the RDP Classifier (Wang *et al*. 2007) and the chloroplast and archaeal sequences were removed. The sequences were deposited in NCBI under bioproject number PRJNA643703. Given that cyanobacteria are primary producers in aquatic ecosystems, as are the diatoms, we separated cyanobacteria from the other components of bacteria (namely, non-cyanobacteria bacteria). Subsequently, cyanobacteria and non-cyanobacteria communities were rarefied at 2800 and 80 000 sequences per sample, respectively, for the following analyses. The following data analyses were performed using all OTUs.

### Data analyses

To test for statistical dependence between the explanatory variables, Spearman's rank (*r_s_*) correlation coefficients were used. Strongly correlated variables (i.e. TN, pool elevation, pool depth and pool area) were excluded so that all pairs of explanatory variables used in data analyses had correlation coefficients *r_s_* ≤ 0.7. As pool volume correlated with pool depth (*r_s_* = 0.80) and area (*r_s_* = 0.96), we chose to use volume as it presumably describes the three-dimensional aquatic ecosystem size more accurately than area. We excluded water temperature because the sampling time and prevailing weather conditions strongly influenced the snapshot temperature measurements.

To assess the degree of spatial autocorrelation in the local environmental variables and microbial richness, we used correlograms with Moran's *I*. Autocorrelation coefficients were calculated for 14 distance classes with ~400 m intervals producing a spatial correlogram. The correlogram was considered significant (*a* ≤ 0.05) if at least one autocorrelation coefficient was significant at a Bonferroni-corrected significance level (*a*/k, where k is the number of distance classes) (Legendre and Legendre 2012).

To examine whether pool salinity influenced the key factors shaping microbial species richness and community composition, the rock pools were divided into freshwater and brackish systems based on their conductivity (used as an estimate for salinity). Pools that had conductivity <1 mS cm^−1^ (salinity ~0.5) were categorized as freshwater systems. In addition, eight pools that had slightly saline water (conductivity 1.14–3.07 mS cm^−1^, salinity ~0.6–1.6) were included in this category because their salinity had presumably only recently increased due to evaporation during the dry summer period. All other pools were categorized as brackish. The number of freshwater pools was 41 and brackish pools 60.

To investigate whether there were significant differences in community compositions between the freshwater and brackish pools, we performed two-dimensional non-metric multidimensional scaling (NMDS) and analysis of similarities (ANOSIM) using Bray-Curtis dissimilarity matrices.

Redundancy analysis (RDA) was used to assess community compositions and their relations to the explanatory variables. For RDA, explanatory variables excluding pH and pool coordinates were log-transformed [log10(x+1)] to reduce their skewed distributions. RDAs were run separately for each microbial group using two subsets of explanatory variables: the environmental variables (conductivity, pH, TP and volume) and the spatial variables (isolation, distance to the sea, latitude and longitude). From the full models, a forward selection of explanatory variables was carried out separately for each subset using function *ordiR2step* with 199 permutations in R package vegan (Oksanen *et al*. 2019). The selected variables were used as explanatory variables in the final RDAs for each microbial group. Multicollinearity among the variables was assessed by variance inflation factors (VIFs); all variables in the final models had VIFs <4. In the RDAs, adjusted *R*^2^ was used to measure the amount of explained variation.

For each microbial group, the forward-selected variables were also used as explanatory variables in variation partitioning to partition the variation in community composition with respect to the environmental variables, spatial variables and their joint effects. The RDAs and variation partitioning were carried out using Hellinger-transformed relative abundance data (Legendre and Gallagher 2001).

To examine the relative effects of water chemistry (conductivity, pH and TP), pool volume and distances to potential sources of immigrants (distance to the sea, distance to the mainland and isolation) on species richness of each microbial group, we used a boosted regression tree (BRT) method. The BRT is an advanced form of regression, which takes into account interactions between explanatory variables and can handle complicated nonlinear functions (Elith, Leathwick and Hastie 2008). We fitted the BRT models using tree complexity = 3, bagging fraction = 0.5 and learning rate = 0.001 with a Gaussian error distribution. Models for freshwater pools were run with bagging fraction = 0.6. All variables were standardized (mean = 0, SD = 1) before they were entered in the BRT models. The BRTs, RDAs, variation partitioning and tests of spatial autocorrelation were performed separately with datasets comprising (i) all pools, (ii) freshwater pools and (iii) brackish pools. In addition, we used simple linear regression to test for the expected positive relationship between species richness and pool size (i.e. pool volume). We also used linear regression to test whether species richness across all pools was positively correlated with island size and negatively correlated with that island's distance to the mainland, calculated as the mean of all pools’ distance to the mainland on each island.

To test for the relationship between environmental heterogeneity and microbial species richness and beta diversity, environmental heterogeneity for each island was estimated as the mean Euclidean distance of standardized (mean = 0, SD = 1) water chemistry variables (conductivity, pH and TP). To assess the effects of individual water chemistry variables, we calculated coefficients of variation (CV) of conductivity, pH and TP (raw values). For each island separately, the total beta diversity of each microbial group was estimated as the total variance in Hellinger-transformed abundance data (function *beta.div* in R package adespatial; Dray *et al*. 2019). The total species richness for each island was calculated as the sum of species observed among the rock pools on the island. To test for the expected positive correlation between beta diversity or species richness and environmental heterogeneity and the CVs, we used linear regression. From the within-island analyses, we excluded island Vindskären, on which only six rock pools were sampled to allow comparison among the islands.

All statistical analyses were performed separately for the data of diatoms, cyanobacteria and non-cyanobacteria, and conducted with R version 3.6.1 (R Core Team 2019) using the packages adespatial (Dray *et al*. 2019), dismo (Hijmans *et al*. 2017), pgirmess (Giraudoux 2018) and vegan (Oksanen *et al*. 2019). The packages ggplot2 (Wickham 2016) and gridExtra (Auguie 2017) were used in figure production.

## RESULTS

The rock pools were typically small and shallow (depth <0.7 m) but varied considerably in other characteristics. For instance, water conductivity ranged from 0.04 to 43.63 mS cm^−1^ (mean: 7.43), pH from 4.9 to 10.8 (mean 8.9) and TP from 28 to 6809 μg L^−1^ (mean: 496). In general, pH was lower in freshwater pools (range: 4.9–10.8; mean: 8.4) than in brackish pools (range: 8–10.3; mean: 9.2). TP concentrations were higher in freshwater pools (range: 30–4296 μg L^−1^; mean: 604) compared with brackish pools (28–6809 μg L^−1^; mean: 422). Distance to the sea varied between 0.3 and 25.7 m (mean: 7.5) and distance to the mainland ranged from 35 to 2912 m.

Among all pools, the most widely distributed diatom species occurring in ≥80% of pools were *Navicula perminuta*, *Tabularia fasciculata*, *Diatoma moniliformis*, *Nitzschia inconspicua* and *Nitzschia microcephala*. *Diatoma moniliformis*, *Na. perminuta* and *T. fasciculata* were widely distributed in both freshwater and brackish pools occurring in >75% of the pools in both ecosystem types, whereas some species showed a clear preference for freshwater or brackish conditions (Table 1). *Achnanthidium minutissimum* and *Pseudostaurosira elliptica* were more common in the freshwater pools, whereas *Berkeleya rutilans* and *Gomphonema olivaceum* were more frequently observed in the brackish pools.

**Table 1.** The most widely distributed diatom species within the freshwater (*n* = 41) and brackish rock pools (*n* = 60). Shown is the percentage of pools where the species occurred and the mean and range of its relative abundance (%) among those pools.

	Pools (%)	Abundance (%)
Species common in freshwater pools		
*Navicula perminuta*	88	5.8 (0.2–45.0)
*Diatoma moniliformis*	80	2.8 (0.2–27.8)
*Tabularia fasciculata*	76	1.2 (0.2–5.5)
*Achnanthidium minutissimum*	68	38.6 (0.2–97.8)
*Nitzschia inconspicua*	66	1.2 (0.2–10.9)
*Pseudostaurosira elliptica*	61	11.8 (0.2–69.0)
*Rhoicosphenia abbreviata*	61	0.8 (0.2–4.1)
*Planothidium delicatulum*	59	0.8 (0.2–3.7)
*Nitzschia microcephala*	56	5.4 (0.2–56.2)
*Nitzschia pusilla*	56	7.6 (0.2–50.8)
Species common in brackish pools		
*Tabularia fasciculata*	100	4.8 (0.2–35.7)
*Navicula perminuta*	98	13.6 (0.2–39.8)
*Nitzschia microcephala*	97	11.2 (0.4–69.6)
*Nitzschia inconspicua*	95	5.4 (0.2–72.5)
*Diatoma moniliformis*	93	10.5 (0.4–42.7)
*Planothidium delicatulum*	93	7.1 (0.2–49.5)
*Berkeleya rutilans*	90	1.0 (0.2–5.1)
*Gomphonema olivaceum*	90	0.9 (0.2–4.8)
*Nitzschia pusilla*	90	14.8 (0.2–83.5)
*Rhoicosphenia abbreviata*	85	2.7 (0.2–62.8)

**Figure 2.**
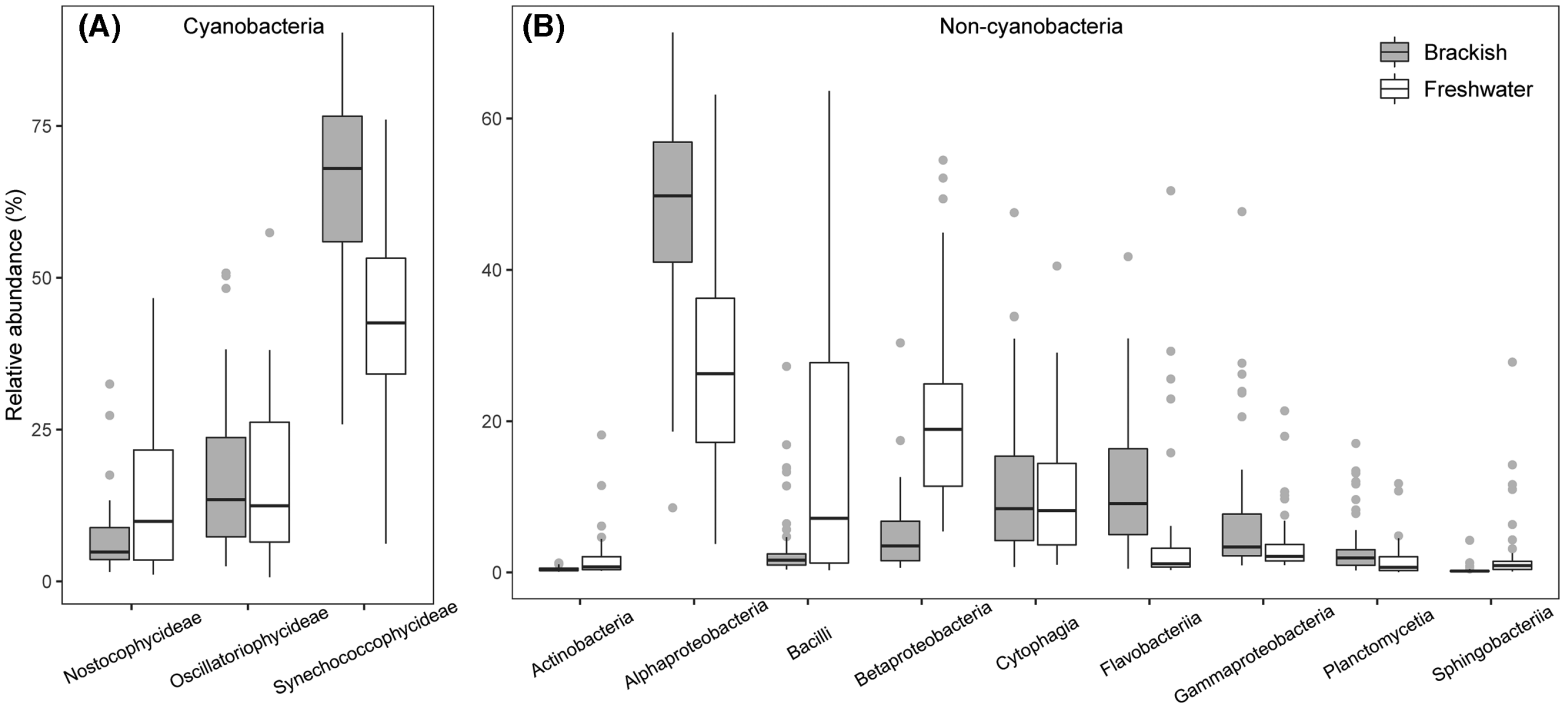
Boxplots showing relative abundances of the most common classes of **(A)** cyanobacteria and **(B)** non-cyanobacteria in brackish and freshwater rock pools in southern Finland.

The most common classes of cyanobacteria were *Nostocophycideae*, *Oscillatoriophycideae* and *Synechococcophycideae* (Fig. 2A). *Synechococcophycideae* was the most abundant class, with markedly higher relative abundances in the brackish pools compared with in the freshwater pools. Among the non-cyanobacterial communities, *Actinobacteria*, *Alphaproteobacteria*, *Bacilli*, *Betaproteobacteria*, *Cytophagia*, *Flavobacteriia*, *Gammaproteobacteria*, *Planctomycetia* and *Sphingobacteriia* were the most common classes among the pools (Fig. 2B). Of these classes, *Alphaproteobacteria* and *Flavobacteriia* were on average clearly more abundant in the brackish pools, whereas *Bacilli* and *Betaproteobacteria* had generally higher relative abundances in the freshwater pools.

Significant spatial autocorrelation was observed for pH and conductivity in all datasets (that is, all pools, freshwater pools and brackish pools) for TP among the freshwater pools and for pool volume among all the pools and the brackish pools (Figs S1–S3 in the supporting information). No indication of strong gradient-like structures was found, however, as the correlograms showed no monotonically decreasing patterns. Diatom richness showed significant spatial autocorrelation among all the pools and among the brackish pools, cyanobacteria richness among all the pools and non-cyanobacteria richness among the freshwater pools (Figs S1–S3). In these cases, the model residuals from the BRTs were tested for spatial autocorrelation; no significant trends were detected.

**Figure 3.**
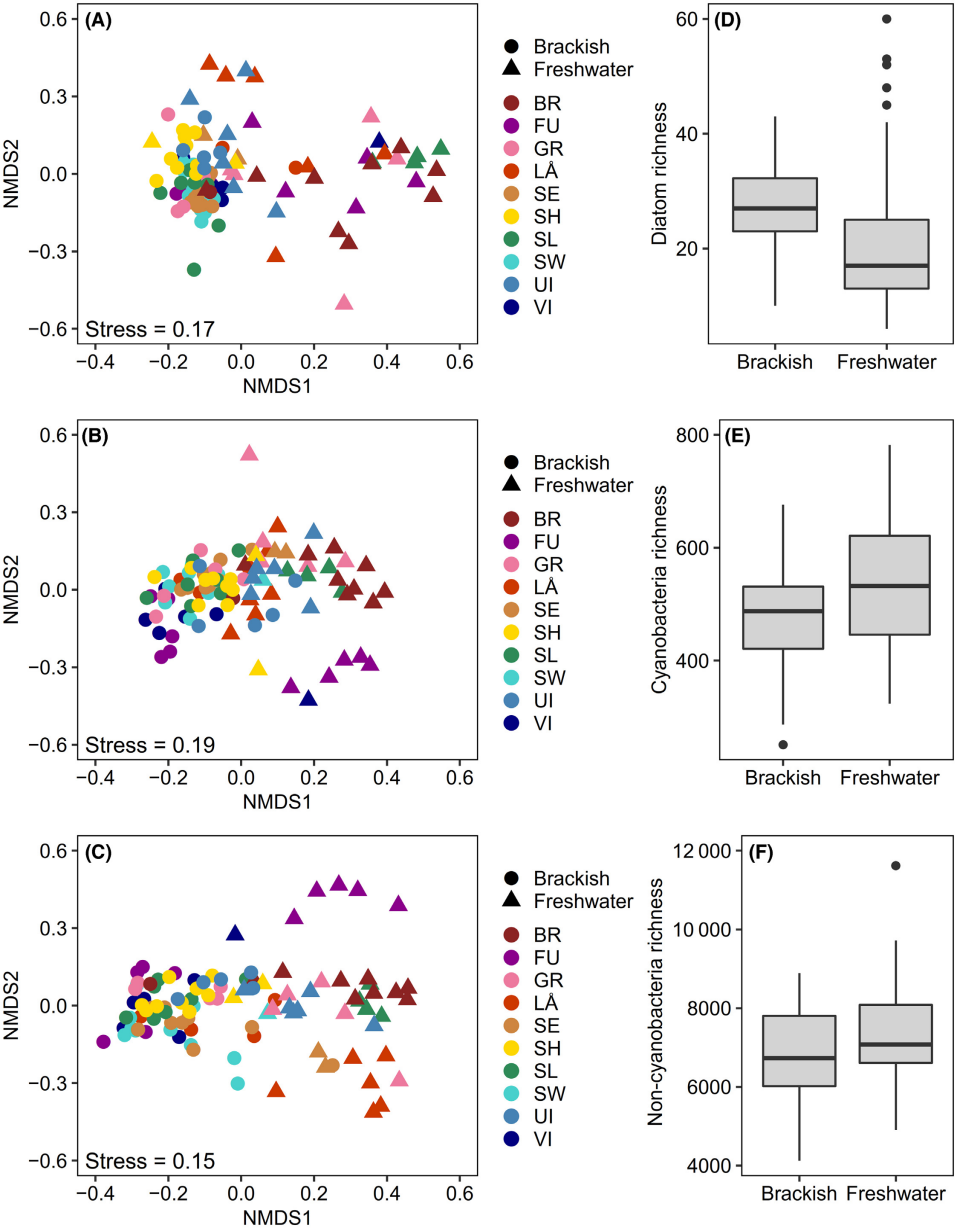
Non-metric multidimensional scaling (NMDS) plots of **(A)** diatom, **(B)** cyanobacteria and **(C)** non-cyanobacteria communities among the rock pools (*n* = 101), and boxplots illustrating species richness of **(D)** diatoms, **(E)** cyanobacteria and **(F)** non-cyanobacteria in brackish (*n* = 60) and freshwater rock pools (*n* = 41) in southern Finland. In the NMDS plots, the colors represent different islands. Abbreviations: BR = Brännskär, FU = Furuskär, GR = Granbusken, LÅ = Långskär, SE = Spikarna eastern island, SH = Storsundsharun, SL = Storlandet, SW = Spikarna western island, UI = Unnamed island, VI = Vindskären.

NMDS revealed that community compositions clearly differed between brackish and freshwater pools and that communities among the brackish pools were more similar to each other than among the freshwater pools (Fig. 3A–C). The results of ANOSIM also showed that brackish and freshwater pools harbored significantly different community compositions (diatoms *R* = 0.49, *P* < 0.001; cyanobacteria *R* = 0.535, *P* < 0.001; non-cyanobacteria *R* = 0.677, *P* < 0.001). For all groups, mean richness values differed significantly between the brackish and freshwater pools (*t*-test, diatoms *P* = 0.01; cyanobacteria *P* = 0.02; non-cyanobacteria *P* = 0.03). Diatom richness was generally higher in brackish pools compared with freshwater pools (Fig. 3D), whereas differences in the richness of cyanobacteria (Fig. 3E) and non-cyanobacteria (Fig. 3F) between the brackish and freshwater pools were smaller.

**Figure 4.**
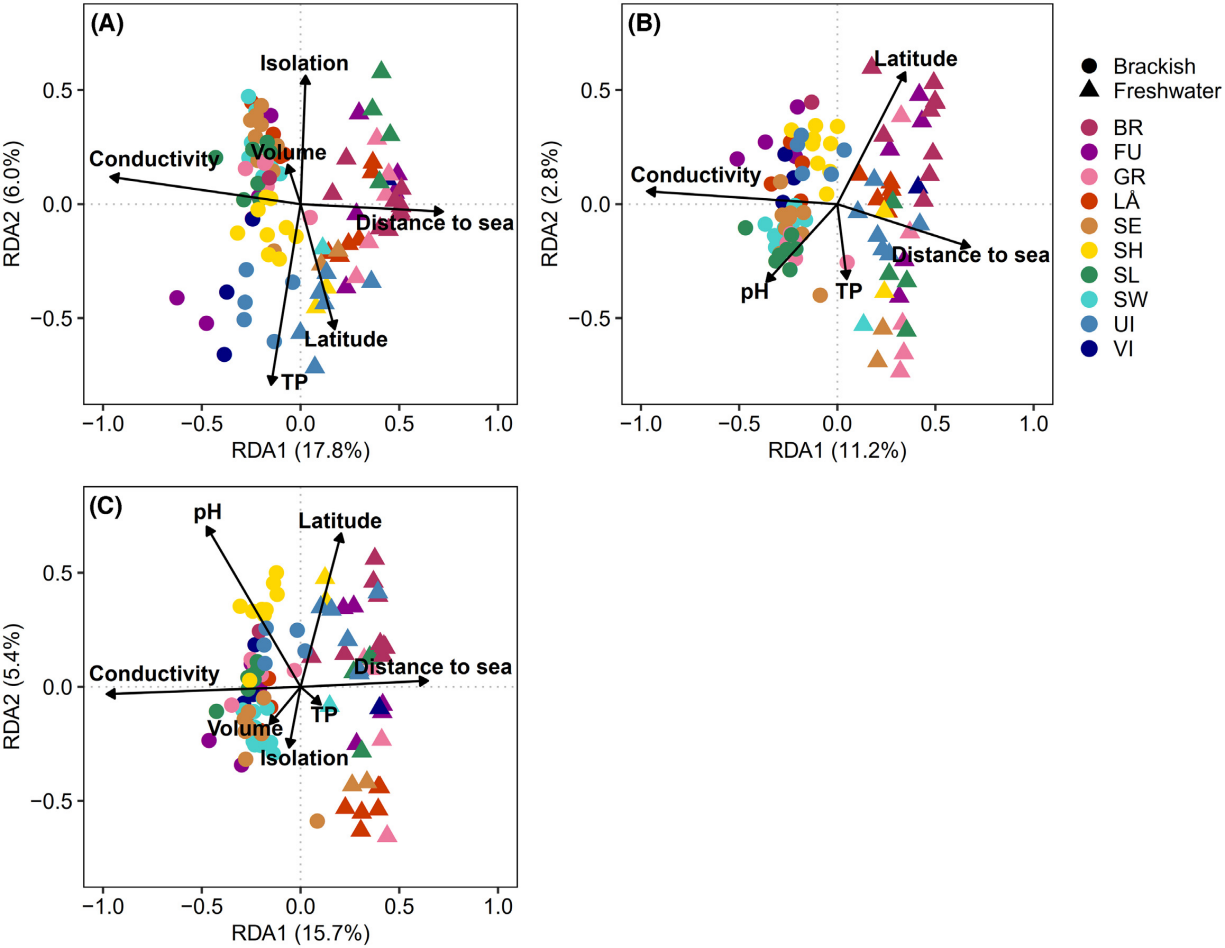
Redundancy analysis (RDA) plots showing the relationships between explanatory variables and community composition of **(A)** diatoms, **(B)** cyanobacteria and **(C)** non-cyanobacteria among rock pools (*n* = 101) in southern Finland. The colors represent different islands; for abbreviations, see Fig. 3.

In RDA comprising all pools, the first axis for each microbial group was most strongly associated with conductivity and distance to the sea (Fig. 4, Table S1). The most important variables along the second axis were TP, isolation and latitude for diatoms (Fig. 4A), latitude for cyanobacteria (Fig. 4B) and pH and latitude for non-cyanobacteria (Fig. 4C). The amount of explained variation (adj. *R^2^*) was 25.0% for diatoms, 14.8% for cyanobacteria and 23.3% for non-cyanobacteria.

Among the freshwater pools, the final RDA for diatoms (adj. *R^2^*= 15.7%) comprised TP and distance to the sea (Table S1). The first axis was most strongly associated with TP. The final RDA for cyanobacteria (adj. *R*^2^ = 9.6%) included TP, pH, latitude and distance to the sea, of which TP was the key variable along the first axis. For non-cyanobacteria, the final RDA (adj. *R*^2^ = 16.9%) included TP, pH, conductivity, latitude and distance to the sea, of which TP and pH were the most important variables.

Among the brackish pools, the final RDA for diatoms (adj. *R*^2^ = 18.9%) comprised conductivity, pH, TP, volume, distance to the sea and latitude; along the first axis, pH, TP and conductivity were most strongly associated with variation in community composition (Table S1). The final model for cyanobacteria (adj. *R*^2^ = 8%) included conductivity, TP, distance to the sea and latitude; conductivity and distance to the sea were the key contributors along the first axis. The final model for non-cyanobacteria (adj. *R*^2^ = 15.9%) included pH, conductivity, TP, volume, latitude, distance to the sea and isolation; the most important variables along the first axis were conductivity and distance to the sea.

Variation partitioning showed that across all pools, community composition of each microbial group was most strongly related to the pure effects of environmental variables and the joint effects of environmental and spatial variables (Table 2). The pure effects of spatial variables were weaker, yet significant (*P* < 0.05). The pure effects of environmental variables also captured the highest fraction of explained variation for all the microbial groups among the freshwater pools and for diatoms and non-cyanobacteria among the brackish pools, while the pure effects of spatial variables covered the highest proportion for cyanobacteria among the brackish pools.

**Table 2.** Results of variation partitioning showing the percentages of explained variation (adj. *R^2^*) in community composition of diatoms, cyanobacteria and non-cyanobacteria among all rock pools (*n* = 101), freshwater rock pools (*n* = 41) and brackish rock pools (*n* = 60). Variation was explained by pure environmental variables (E|S), pure spatial variables (S|E) and their joint effects (E∩S). Statistically significant values of the testable fractions (E|S and S|E) are shown in bold. Res. = unexplained variation.

	E|S	S|E	E∩S	Res.
All rock pools				
Diatoms	**11.8**	**4.1**	9.0	75.0
Cyanobacteria	**6.8**	**2.8**	5.2	85.2
Non-cyanobacteria	**13.1**	**3.1**	7.1	76.7
Freshwater rock pools				
Diatoms	**11.6**	**2.0**	2.2	84.3
Cyanobacteria	**5.0**	**3.3**	1.2	90.4
Non-cyanobacteria	**10.6**	**2.5**	3.8	83.1
Brackish rock pools				
Diatoms	**8.3**	**3.9**	6.8	81.1
Cyanobacteria	**2.4**	**5.0**	0.6	92.0
Non-cyanobacteria	**9.1**	**5.8**	1.0	84.1

**Figure 5.**
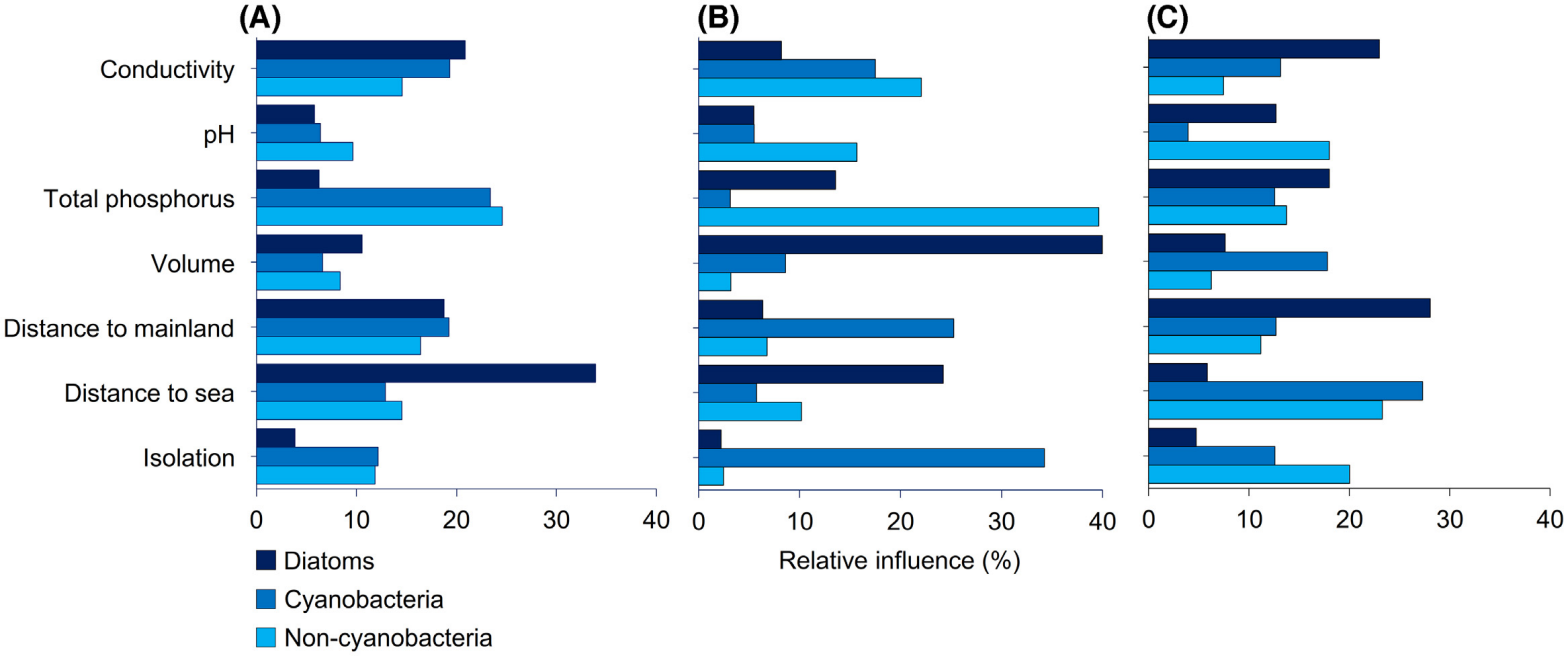
Relative contributions of explanatory variables in boosted regression tree analyses to explain the variation in species richness of diatoms, cyanobacteria and non-cyanobacteria among **(A)** all rock pools (*n* = 101), **(B)** freshwater rock pools (*n* = 41) and **(C)** brackish rock pools (*n* = 60) in southern Finland.

According to the BRT analyses, distance to the sea had the highest relative influence on diatom richness and TP on cyanobacteria and non-cyanobacteria richness when all pools were considered (Fig. 5A, Fig. S4). Among the freshwater pools, pool volume had the highest relative influence on diatom richness, isolation on cyanobacteria richness and TP on non-cyanobacteria richness (Fig. 5B, Fig. S5). Among the brackish pools, distance to the mainland was the strongest contributor to diatom richness and distance to the sea on cyanobacteria and non-cyanobacteria richness (Fig. 5C, Fig. S6). In linear regression, no significant relationships between species richness and pool volume were found (*P* = ns for all microbial groups).

Based on linear regression, there was a negative relationship between diatom richness and island size (*R*^2^ = 0.05, *P* = 0.03) and distance to the mainland (*R*^2^ = 0.09, *P* = 0.003), whereas cyanobacteria and non-cyanobacteria richness exhibited no significant trends with island size or distance to the mainland (Fig. S7).

Within islands, beta diversity was positively correlated with environmental heterogeneity for all the groups; the relationship was significant for cyanobacteria (*R^2^* = 0.54, *P* = 0.02) and non-cyanobacteria (*R^2^* = 0.50, *P* = 0.03), but not for diatoms (*R^2^* = 0.23, *P* = ns). Beta diversity for all the groups was positively associated with CV of pH (diatoms *R^2^* = 0.67, *P* = 0.007; cyanobacteria *R^2^* = 0.44, *P* = 0.05; non-cyanobacteria *R^2^* = 0.50, *P* = 0.03). Diatom beta diversity was also positively correlated with CV of conductivity (*R^2^* = 0.49, *P* = 0.04). No significant correlations between within-island richness and environmental heterogeneity or CVs of the individual water chemistry variables were found.

## DISCUSSION

To our knowledge, this is the first study to examine multiple microbial groups among and within islands in a set of rock pools that provides an excellent model system to gain insights into the roles of local and regional processes shaping biodiversity patterns. Although microorganisms are key players in various ecosystem functions, considerable knowledge gaps regarding their biodiversity patterns and determinants remain. Here, we examined the degree to which local environmental and spatial factors influence microbial community compositions and tested whether variation in species richness is best explained by water chemistry or by pool size and distances to source pools, thus complying with the theory of island biogeography.

The ordination results revealed that across all pools, community compositions clearly changed along the first RDA axis. For each microbial group, the axis correlated primarily with conductivity and distance to the sea, representing thus the transition from brackish pools under a stronger marine influence towards freshwater pools maintained mainly by precipitation in the more sheltered interior parts of the islands. This outcome is in line with earlier studies, in which salinity or conductivity had been identified as key factors influencing microbial community composition in a variety of ecosystems, such as rock pools (Aarnio, Teittinen and Soininen 2019), lakes (Wang *et al*. 2011) and brackish systems (Ulanova, Busse and Snoeijs 2009; Herlemann *et al*. 2011). Along with conductivity, pool distance to the sea contributed to the segregation of communities along the first axis, suggesting that not only species-sorting mechanisms, but also dispersal-related factors, play a role in community assembly. Rock pools closer to the sea are more likely to receive occasional inputs of seawater and associated colonization by microbes, subsequently leading to more similar communities along the seashores.

The RDA results were consistent with those of NMDS and ANOSIM, indicating that brackish and freshwater pools differed in terms of community composition. Among the common diatom species in these data, freshwater species *A. minutissimum* and *P. elliptica* were more often observed in the freshwater pools, frequently occurring in high relative abundances. *Achnanthidium minutissimum* is a very common species, often found in high abundances in a variety of freshwater habitat types due to its apparently wide ecological amplitude (Cantonati, Kelly and Lange-Bertalot 2017). *Berkeleya rutilans* and *G. olivaceum* were more widely distributed in the brackish pools. *Berkeleya rutilans* is a common species in brackish systems (Busse and Snoeijs 2003; Ulanova, Busse and Snoeijs 2009), thriving in, for instance, coastal regions or saline lakes (Cantonati, Kelly and Lange-Bertalot 2017). In the Baltic Sea, *G. olivaceum* has been found to have a preference for high exposure to wave action (Busse and Snoeijs 2003), which may in part explain its wider distribution among the more exposed brackish pools closer to the sea. Three species (i.e. *Na. perminuta*, *D. moniliformis* and *T. fasciculata*) were widely distributed in both freshwater and brackish pools, but their relative abundances were on average higher in the brackish pools compared with the freshwater pools. *Navicula perminuta* and *T. fasciculata* have been found to occur along a wide salinity range, with increasing relative abundances towards higher salinities also in the Baltic Sea area (Ulanova, Busse and Snoeijs 2009). Likewise, *D. moniliformis* has an apparently wide salinity tolerance as it is common in the Baltic Sea (Virta and Soininen 2017), but also found in freshwater ecosystems with elevated conductivity (Krammer and Lange-Bertalot 1991a).

Among cyanobacterial communities, the changing community composition along the freshwater–brackish transition is in line with the results of Székely and Langenheder (2014), who show that conductivity is the key determinant of cyanobacteria community composition in a coastal rock pool system. They did not, however, present the groups within cyanobacteria that contributed to the differentiation along the conductivity gradient. In the present study, *Synechococcophycideae* had the overall highest relative abundances of the most common classes and recorded notably higher abundances in the brackish than in the freshwater systems. Members of this group have also been found to exhibit abundance shifts along the Baltic Sea salinity gradient (Herlemann *et al*. 2011, 2016). Among the non-cyanobacterial communities, the relative abundance of *Alphaproteobacteria* was generally notably higher in the brackish pools compared with the freshwater pools while *Betaproteobacteria* showed an opposing trend. Similarly, these groups are among the dominating bacterial groups in a Baltic Sea study, with increasing relative abundance of *Alphaproteobacteria* and decreasing relative abundance of *Betaproteobacteria* with increasing salinity (Herlemann *et al*. 2011). *Alphaproteobacteria* and *Betaproteobacteria* are also found to be among the most common bacterial groups in rock pools of the Swedish coastal region and conductivity is identified as the most important environmental factor influencing their community composition (Székely and Langenheder 2014). *Flavobacteriia*, which have been reported also as abundant and important members of coastal marine biofilms (Pollet *et al*. 2018), were relatively more common in the brackish pools than freshwater pools. Yet another class clearly contributing to the differentiation between the freshwater and brackish systems was *Bacilli*, members of which showed higher relative abundances in the freshwater pools.

When brackish and freshwater pools were analyzed separately, differences in the relative importance of variables driving community composition emerged. Among the brackish pools, conductivity and distance to the sea continued to be the main factors affecting cyanobacteria and non-cyanobacteria, while diatom community composition was most strongly associated with pH along the first axis. The lesser influence of conductivity for the diatoms is rather unexpected given that there was a quite long conductivity gradient, even within the brackish pools. This finding may reflect the fact that most of the common diatom species observed among the brackish pools showed wide distributions and are thus presumably tolerant to a wide conductivity range. The differing outcome between the diatoms and bacterial communities could also be perhaps because of differing methods to obtain the species data between these groups, that is, molecular versus morphological identification. The morphological diatom identification yielded lower levels of diatom diversity compared with the bacterial communities, and the challenges in identifying some fine-scale morphological features consistently under the light microscope may have limited the taxonomic resolution and hence resulted in overestimation of some diatom species tolerances towards conductivity.

Among the freshwater pools, variation in community composition of each microbial group was most strongly related to TP, implying that this variable imposes a strong selective filter among these pools, leading to greater similarity in community compositions in pools with more similar phosphorus concentrations. This outcome disagrees with a study where neither environmental nor spatial factors could explain bacterial community composition among freshwater pools, leading the authors to suggest that as rock pools provide both spatially and temporally highly heterogeneous habitats, they may sustain communities comprising species that are generalists with respect to environmental factors other than conductivity (Székely and Langenheder 2014). Such contrasting findings compared with ours may, however, stem from the differences in study extent and the observed microbial diversity as their study was conducted on a single island with fewer pools and lower levels of bacterial diversity than the present study.

In general, the main mechanism underlying changes in community composition appears to be species sorting as pure effects of environmental variables typically captured the highest proportion of explained community variation across microbial groups and pool types. Species sorting by local environmental conditions often has a strong imprint on community composition in heterogeneous environments where dispersal rates are sufficient to sort species into localities based on their environmental characteristics (Heino *et al*. 2015). Species sorting has also often been reported as the leading mechanism affecting microbial communities (Van der Gucht *et al*. 2007; Souffreau *et al*. 2015; Rodríguez-Alcalá *et al*. 2020). The effects of pure spatial factors were, however, also significant for all microbial groups regardless of whether all pools, only freshwater pools or only brackish pools were considered, suggesting that dispersal-related processes influence the structuring of communities. This finding complies with earlier evidence on the importance of spatial factors on aquatic microbial communities, even at relatively small spatial extents (Langenheder and Ragnarsson 2007; Aarnio, Teittinen and Soininen 2019). Deciphering whether the spatial component implies high dispersal rates, that is, mass effects (Shmida and Wilson 1985) or dispersal limitation, is not straightforward as both mechanisms may obscure species sorting by either homogenizing community compositions of nearby sites or by preventing species from occurring at all suitable localities. Typically, however, mass effects are considered more prevalent at small spatial extents, whereas dispersal limitation is more likely at relatively large spatial scales (Cottenie *et al*. 2003; Langenheder and Lindström 2019).

The BRTs emphasized the effects of both environmental and spatial factors in driving species richness, but the relative influence of the explanatory variables differed among the microbial groups and pool types. Whereas changes in community composition across the pools were tightly linked to conductivity, other factors had higher relative effects on richness, complying with earlier studies reporting less pronounced patterns in microbial richness compared with species composition along salinity gradients (Herlemann *et al*. 2011; Wang *et al*. 2011). Across all pools, diatom richness was primarily associated with pool distance to the sea. Richness decreased with increasing distance to the sea, which may reflect more frequent colonization events from the sea facilitating species coexistence in pools closer to the sea. Diatom richness in the relatively more isolated freshwater pools was also generally lower compared with brackish pools, lending further support for the importance of distance to potential source pools on the number of species, as predicted by the theory of island biogeography (MacArthur and Wilson 1967). This finding could, though, also indicate the effects of unmeasured variables (e.g. grazing pressure) covarying with pool distance to the sea. The secondary importance of local environmental variables on diatom richness disagrees with Virta and Soininen (2017), who found that diatom richness in the Baltic Sea was best explained by pH and nutrients. In contrast to diatoms, variation in both cyanobacteria and non-cyanobacteria richness across the pools was most strongly related to TP, agreeing with earlier studies on the importance of nutrient availability on bacterial richness (Wang *et al*. 2017). The differences in the relative importance of the driving variables were so distinct between the groups that it is unlikely that they could be attributed to the different methods in obtaining the species data; rather, they are possibly linked to differences in dispersal ability among the groups. Bacteria may be more efficient dispersers (Wang *et al*. 2012) and their diversity across the fragmented rock pool system thus less limited by distance to source pools compared with the diatoms.

When only freshwater pools were considered, TP continued to have the highest relative influence on non-cyanobacteria richness. Compared with the non-cyanobacteria, water chemistry variables had weaker effects on the richness of autotrophs, that is, diatoms and cyanobacteria, despite a long gradient in, for instance pH, which has often been reported as being a key variable for aquatic primary producers (Teittinen *et al*. 2017; Wang *et al*. 2017). This outcome suggests that the diversity of diatoms and cyanobacteria among the freshwater systems is less sensitive to changes in local environmental conditions compared with that of non-cyanobacteria. It should be noted, though, that direct comparison of the results among the three taxonomic groups is hindered by differences in ecological diversity as the non-cyanobacteria comprise multiple phyla, the diversity of which may differ in their responses to environmental gradients (Yeh *et al*. 2019). Interestingly, cyanobacteria richness showed the strongest association with pool isolation. Less isolated pools tended to have a higher species richness, suggesting that with decreasing distance to neighboring pools, colonization becomes more likely, increasing the number of species. Previous studies conducted on rock pool invertebrates have shown, for instance, that the number of neighboring pools may facilitate regional coexistence (Hanski and Ranta 1983) and that pool isolation may reduce species richness (Vanschoenwinkel, Buschke and Brendonck 2013). Such a strong influence of isolation was, however, rather unexpected for a group of microorganisms with efficient dispersal capacity. Notably, we found no support for a positive relationship between pool volume and species richness in these data. Diatom richness in freshwater pools was most strongly associated with pool volume but small pools tended to have higher species richness than larger pools. This could be due to lower grazing pressure compared with larger pools that are known to harbor larger populations of, for instance, aquatic crustaceans of the genus *Daphnia* (Ebert, Hottinger and Pajunen 2001). In addition to pool size, none of the microbial groups showed a positive relationship between species richness and size of the island.

Among the brackish pools, variation in species richness of all microbial groups was primarily associated with spatial variables. For diatoms, richness decreased with increasing distance to the mainland. Given that the Baltic Sea likely serves as the main source of immigrants to the brackish pools, this outcome may imply the effects of disturbances, rather than colonization dynamics, in influencing richness. As islands farther from the mainland typically have higher wind exposure (Korvenpää, Von Numers and Hinneri 2003), their coastal rock pools are likely to be subject to more frequent and intense wind- and wave-induced disturbances compared with pools on more sheltered islands closer to the mainland, subsequently leading to impoverished communities at high disturbance intensity and frequency (Sousa 1979; L. Virta, pers. comm.). Disturbances have also been shown to affect bacterial communities (Berga, Székely and Langenheder 2012) and could potentially explain why the richness of both cyanobacteria and non-cyanobacteria tended to increase with increasing distance to the sea, that is, towards the relatively more stable pools farther from the sea.

The overall relatively high proportion of unexplained variation in the analyses of species richness and composition may stem from the effects of unmeasured factors, such as physical disturbances and biotic interactions. The unexplained variation may also be attributable to the snapshot sampling used or stochasticity in microorganisms’ occurrences (Soininen, Korhonen and Luoto 2013). Moreover, although spatial factors were used to indicate dispersal-related processes, the extent to which they capture true dispersal effects here remains uncertain.

Within islands, the beta diversity of all microbial groups was positively correlated with different measures of spatial variation in environmental conditions, that is, environmental heterogeneity or variability in pH or conductivity. However, species richness showed no correlations with these variables, indicating that longer environmental gradients and diversity of habitat types within an island tended to increase variation in community composition among the pools, but such variability was not linked to patterns in richness. Such decoupling of the two measures of biodiversity suggests that changes in community composition among sites were primarily due to changes in species identities and abundances reflecting turnover as opposed to differences in richness; hence, changes in species composition may reflect changing environmental conditions more accurately than trends in species richness (Hillebrand *et al*. 2018). The extent to which microorganisms generally conform to the relationship between environmental heterogeneity and beta diversity remains contested, though, as previous studies have yielded contrasting results (Jyrkänkallio‐Mikkola, Heino and Soininen 2016; Zorzal-Almeida, Bini and Bicudo 2017), indicating context dependency in the observed patterns.

In conclusion, this study increased our knowledge about the factors influencing microbial biodiversity by exploring three taxonomic groups with broadly different functional roles in rock pool systems on islands of the Baltic Sea. Community compositions differed sharply between the brackish and freshwater pools, presumably due to species sorting coupled with dispersal-related processes at the transition zone connecting the sea and rock pool systems. The factors underlying community assembly differed when the brackish and freshwater pools were analyzed separately, highlighting the importance of sampling extent and the length of environmental gradients in mediating the observed community patterns. The relative influence of factors explaining species richness also differed depending on which dataset and microbial group was examined, suggesting that the patterns are context-dependent. Multiple aspects of microbial richness were primarily linked to nutrient availability or distances to potential source pools, whereas no support for a positive relationship between richness and ecosystem size was found. Within islands, environmental heterogeneity increased beta diversity, but not species richness, indicating that beta diversity here reflects species turnover, not species gains or losses.

## Supplementary Material

fiab049_Supplemental_Files
