# Supplementary material for: Factors influencing the biodiversity of three microbial groups within and among islands of the Baltic Sea
Source: FEMS Microbiol Ecol. 2021 Mar 22;97(5):fiab049. doi: 10.1093/femsec/fiab049 (PMC8044292; doi:10.1093/femsec/fiab049)
Supplement: fiab049_Supplemental_Files [file fiab049_supplemental_files.zip › FEMS_Manuscript_Revised_SuppInfo.docx]

**SUPPORTING INFORMATION**

**Table S1**. Results of redundancy analysis for community composition of diatoms, cyanobacteria and non-cyanobacteria and explanatory variables among all pools (n = 101), freshwater pools (n = 41), and brackish pools (n = 60). **P* < 0.05, ***P* < 0.01, ns = not significant. TP = total phosphorus, volume = pool water volume.

|  | Variable | RDA 1 | RDA 2 | Pr (>F) |
| --- | --- | --- | --- | --- |
| **All pools** |  |  |  |  |
| Diatoms | Conductivity | -0.963 | 0.120 | ** |
|  | TP | -0.149 | -0.792 | ** |
|  | Volume | -0.065 | 0.173 | * |
|  | Distance to sea | 0.721 | -0.033 | * |
|  | Latitude | 0.173 | -0.548 | * |
|  | Isolation | 0.025 | 0.563 | ** |
| Cyanobacteria | Conductivity | -0.969 | 0.057 | ** |
|  | pH | -0.361 | -0.341 | ** |
|  | TP | 0.048 | -0.328 | * |
|  | Distance to sea | 0.676 | -0.188 | * |
|  | Latitude | 0.346 | 0.579 | ** |
| Non-cyanobacteria | Conductivity | -0.982 | -0.032 | ** |
|  | pH | -0.475 | 0.703 | ** |
|  | TP | 0.103 | -0.078 | ** |
|  | Volume | -0.157 | -0.163 | ** |
|  | Distance to sea | 0.645 | 0.027 | * |
|  | Latitude | 0.205 | 0.673 | ** |
|  | Isolation | -0.058 | -0.266 | * |
| **Freshwater pools** |  |  |  |  |
| Diatoms | TP | -0.968 | 0.253 | ** |
|  | Distance to sea | 0.497 | 0.868 | * |
| Cyanobacteria | pH | -0.293 | 0.955 | ** |
|  | TP | -0.817 | 0.195 | ** |
|  | Latitude | 0.393 | 0.464 | * |
|  | Distance to sea | 0.471 | -0.111 | * |
| Non-cyanobacteria | pH | -0.477 | 0.796 | ** |
|  | TP | 0.507 | 0.650 | ** |
|  | Conductivity | 0.269 | 0.749 | * |
|  | Latitude | -0.603 | 0.283 | ns |
|  | Distance to sea | -0.107 | -0.535 | ns |
| **Brackish pools** |  |  |  |  |
| Diatoms | pH | 0.547 | -0.033 | ** |
|  | Conductivity | -0.374 | 0.338 | ** |
|  | TP | 0.405 | 0.460 | ** |
|  | Volume | -0.029 | -0.976 | ** |
|  | Distance to sea | 0.258 | -0.296 | * |
|  | Latitude | 0.781 | 0.318 | * |
| Cyanobacteria | Conductivity | -0.598 | -0.324 | ** |
|  | TP | -0.033 | 0.456 | * |
|  | Distance to sea | 0.755 | -0.567 | ** |
|  | Latitude | 0.182 | 0.894 | * |
| Non-cyanobacteria | pH | 0.163 | -0.909 | ** |
|  | Conductivity | -0.573 | -0.122 | ** |
|  | Volume | 0.001 | -0.274 | * |
|  | TP | 0.201 | 0.103 | * |
|  | Latitude | 0.454 | -0.363 | * |
|  | Distance to sea | 0.496 | 0.115 | ** |
|  | Isolation | -0.575 | 0.151 | ns |

**
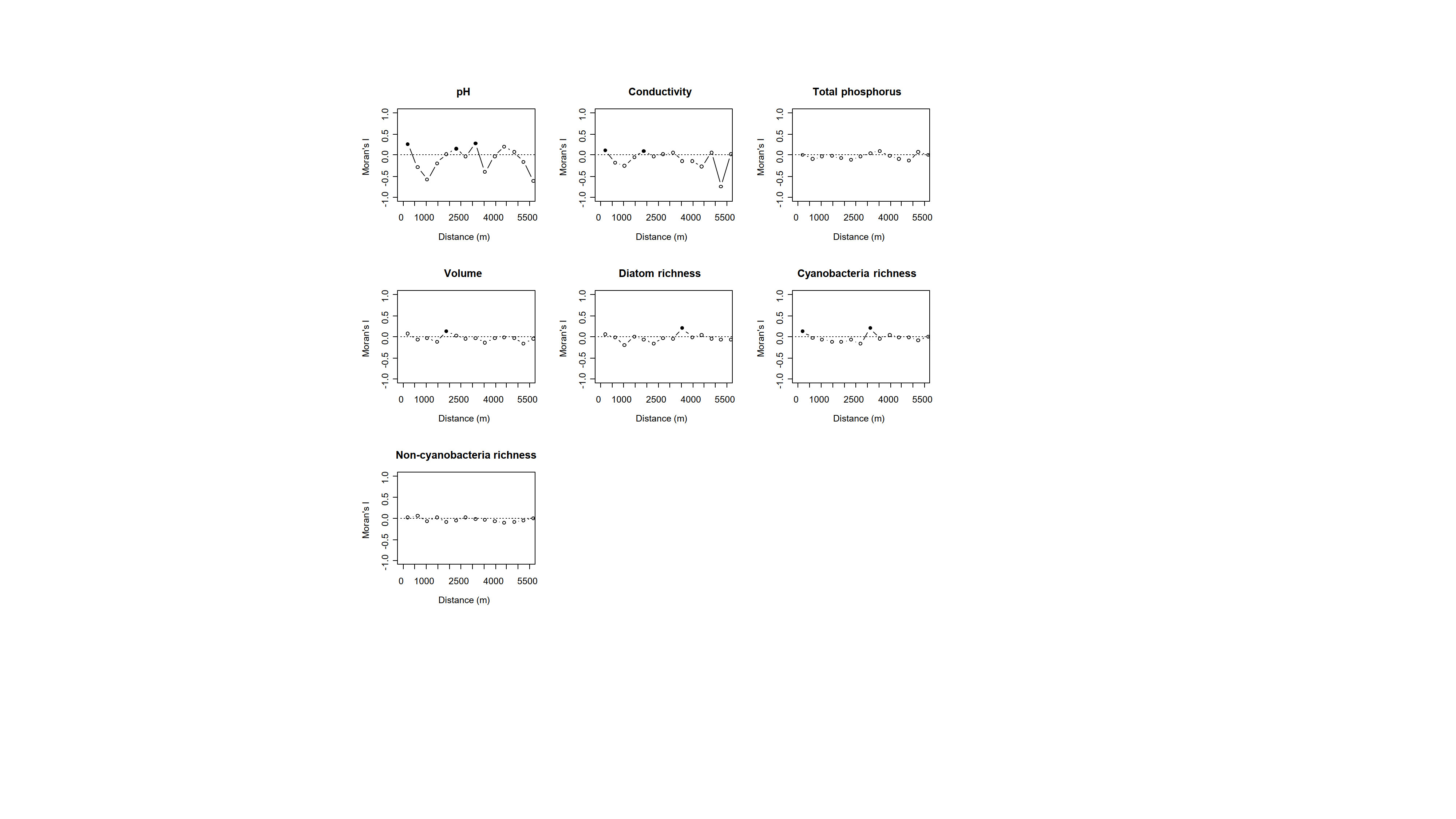
**

**Figure S1**. Spatial autocorrelation in local environmental variables and microbial species richness among all rock pools (n = 101) based on Moran’s *I*. Filled circles indicate autocorrelation coefficients remaining significant after Bonferroni correction at P = 0.05/14 = 0.0036.

**
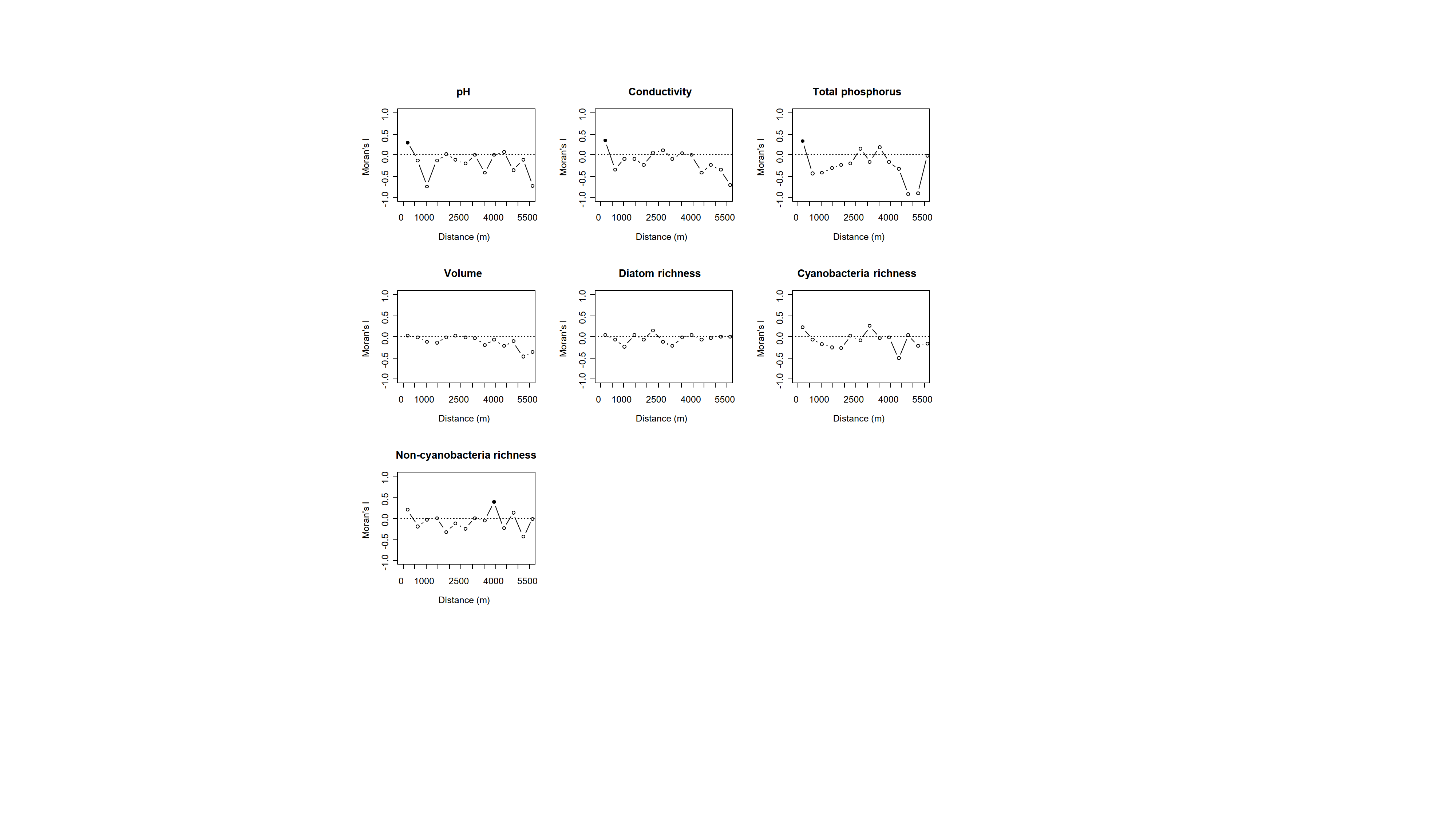
**

**Figure S2**. Spatial autocorrelation in local environmental variables and microbial species richness among freshwater rock pools (n = 41) based on Moran’s *I*. Filled circles indicate autocorrelation coefficients remaining significant after Bonferroni correction at P = 0.05/14 = 0.0036.


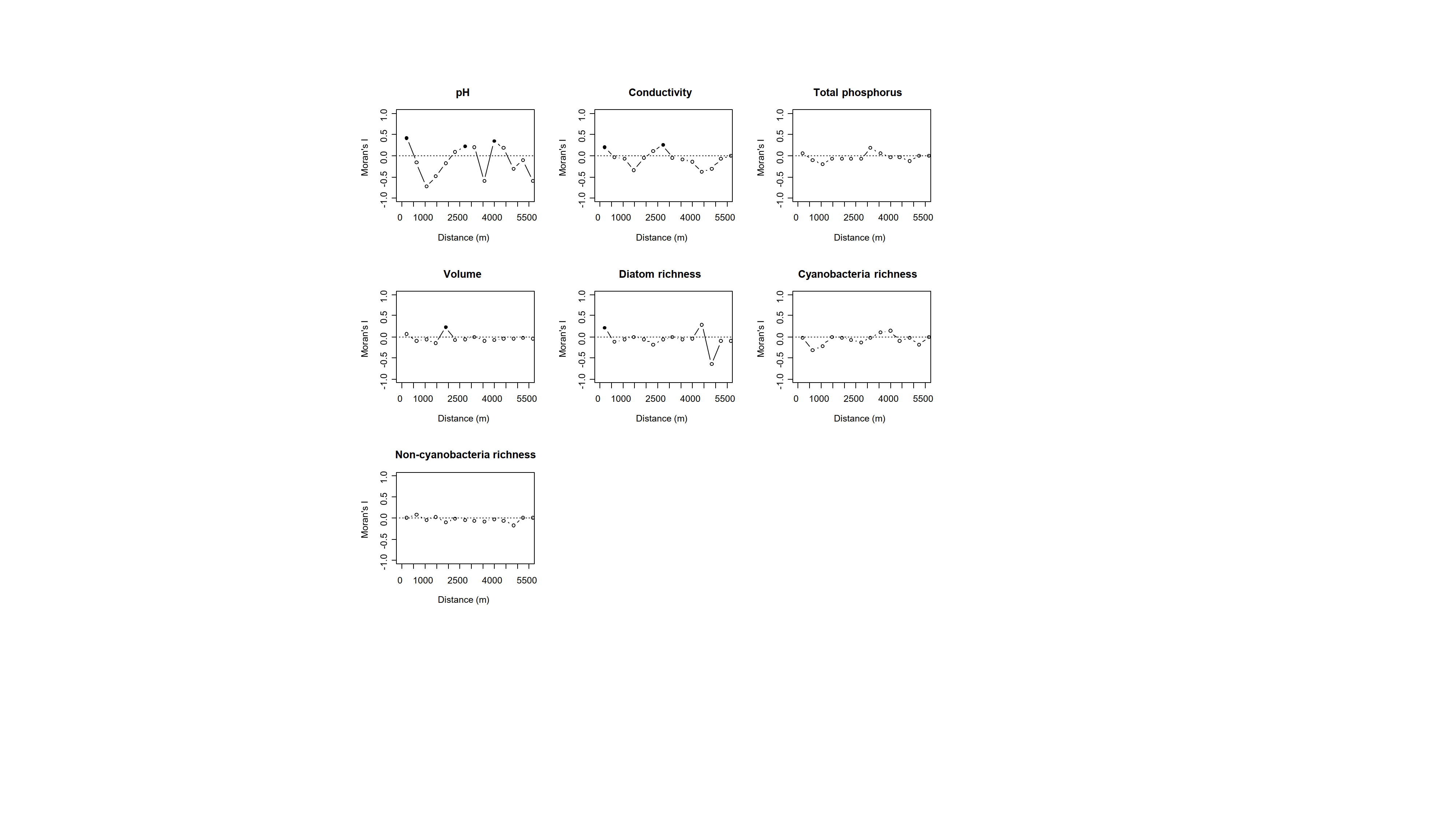


**Figure S3**. Spatial autocorrelation in local environmental variables and microbial species richness among brackish rock pools (n = 60) based on Moran’s *I*. Filled circles indicate autocorrelation coefficients remaining significant after Bonferroni correction at P = 0.05/14 = 0.0036.


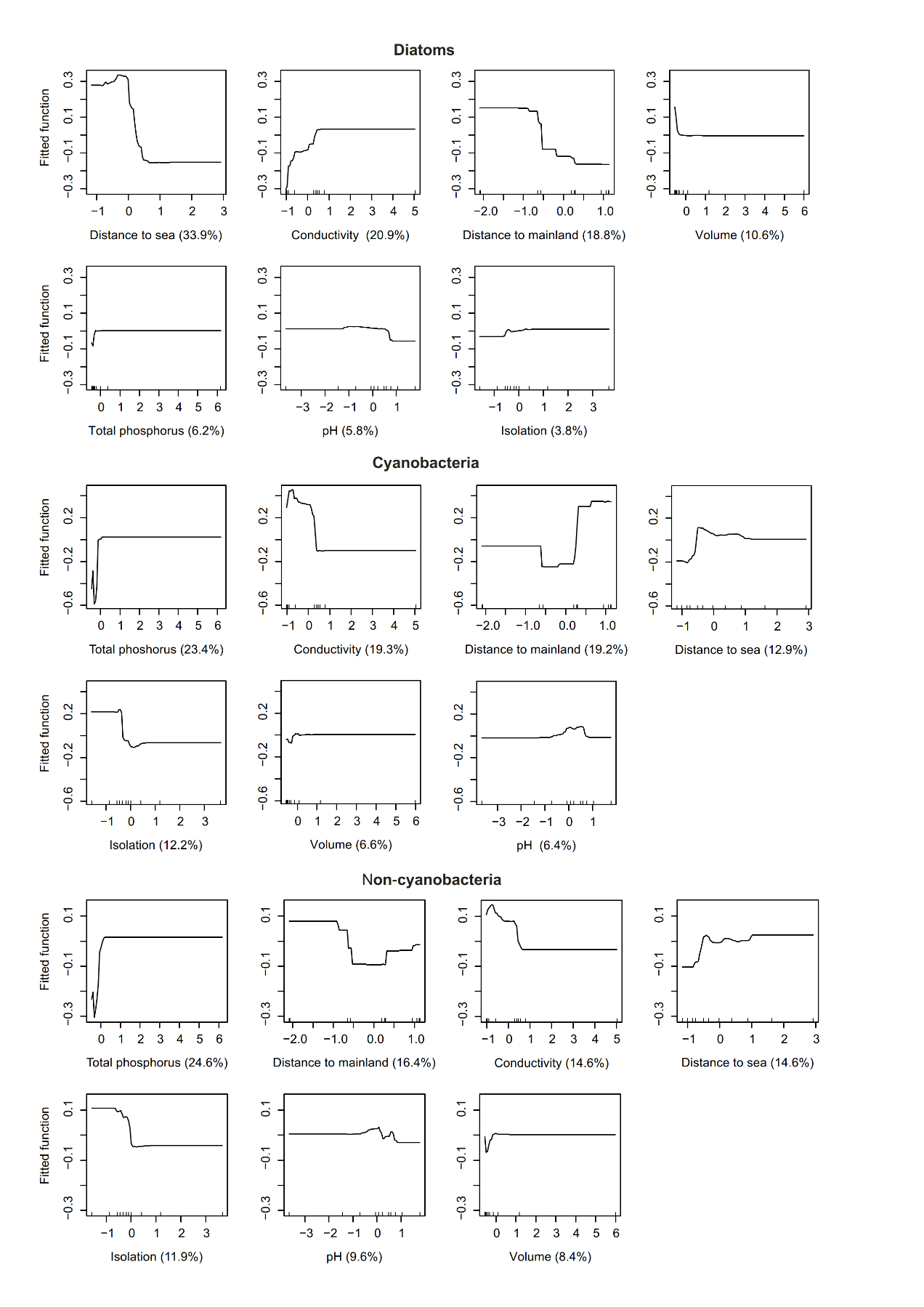


**Figure S4**. Partial dependence plots of explanatory variables used in boosted regression tree analyses for species richness of diatoms, cyanobacteria, and non-cyanobacteria among all rock pools (n = 101). The plots show the effect of an explanatory variable on the response variable after accounting for the average effects of the model's all other variables. Explained deviance of the model was 35.3% for diatoms, 53.9% for cyanobacteria, and 24.5% for non-cyanobacteria.


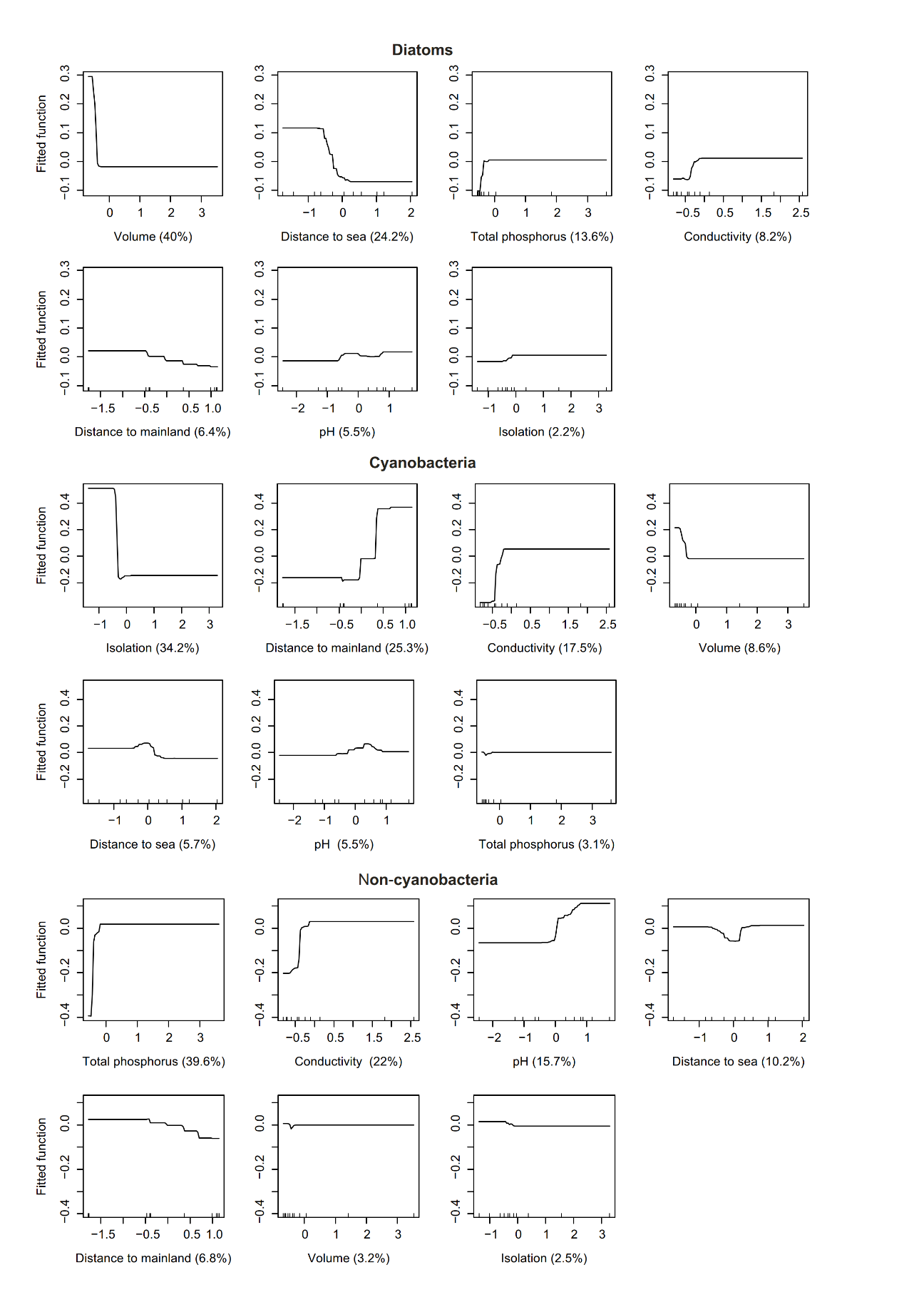


**Figure S5**. Partial dependence plots of explanatory variables used in boosted regression tree analyses for species richness of diatoms, cyanobacteria, and non-cyanobacteria among the freshwater rock pools (n = 41). The plots show the effect of an explanatory variable on the response variable after accounting for the average effects of the model's all other variables. Explained deviance of the model was 19.6% for diatoms, 48.0% for cyanobacteria, and 28.1% for non-cyanobacteria.


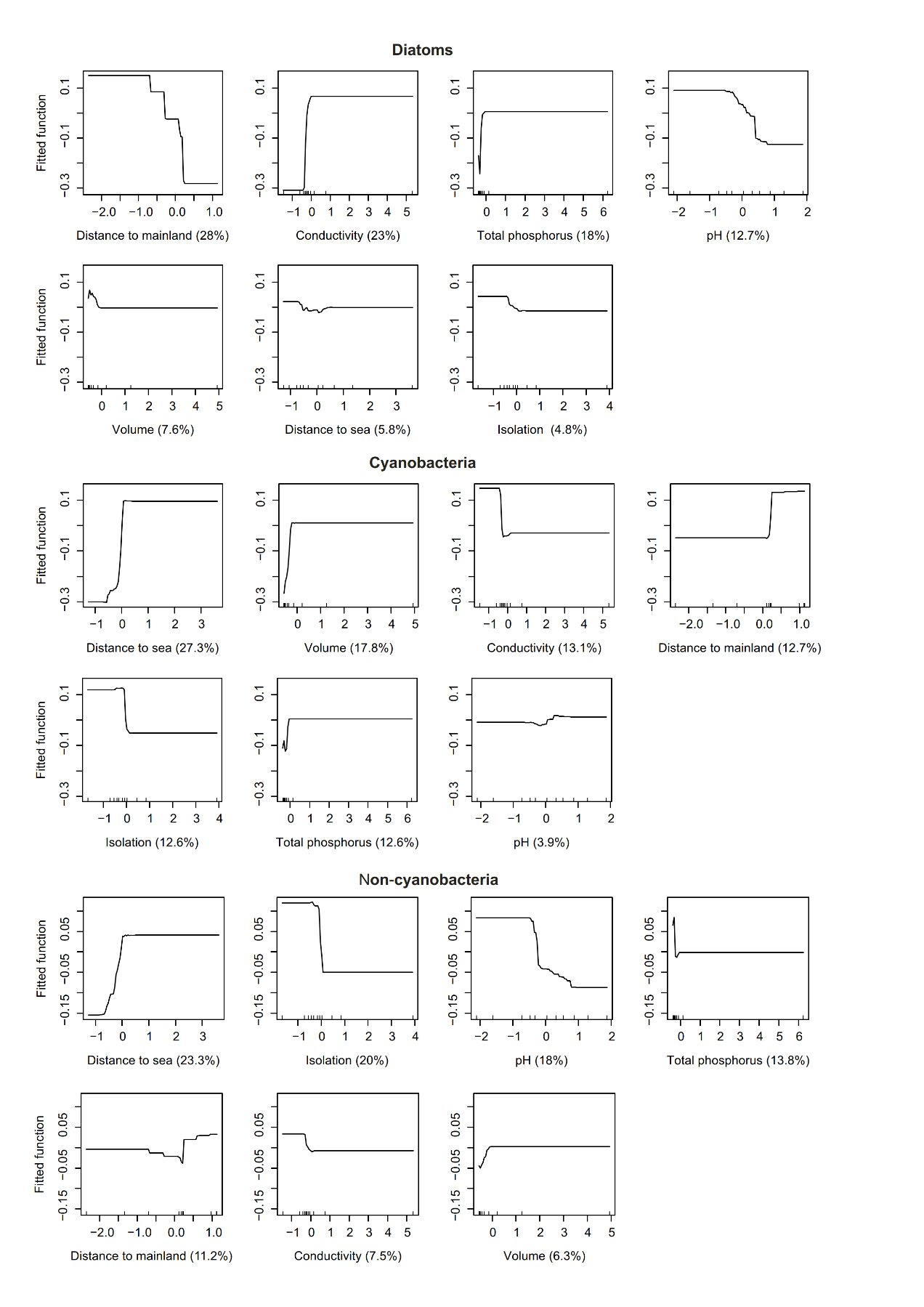


**Figure S6**. Partial dependence plots of explanatory variables used in boosted regression tree analyses for species richness of diatoms, cyanobacteria, and non-cyanobacteria among the brackish rock pools (n = 60). The plots show the effect of an explanatory variable on the response variable after accounting for the average effects of the model's all other variables. Explained deviance of the model was 29.1% for diatoms, 31.4% for cyanobacteria, and 16.1% for non-cyanobacteria.

**
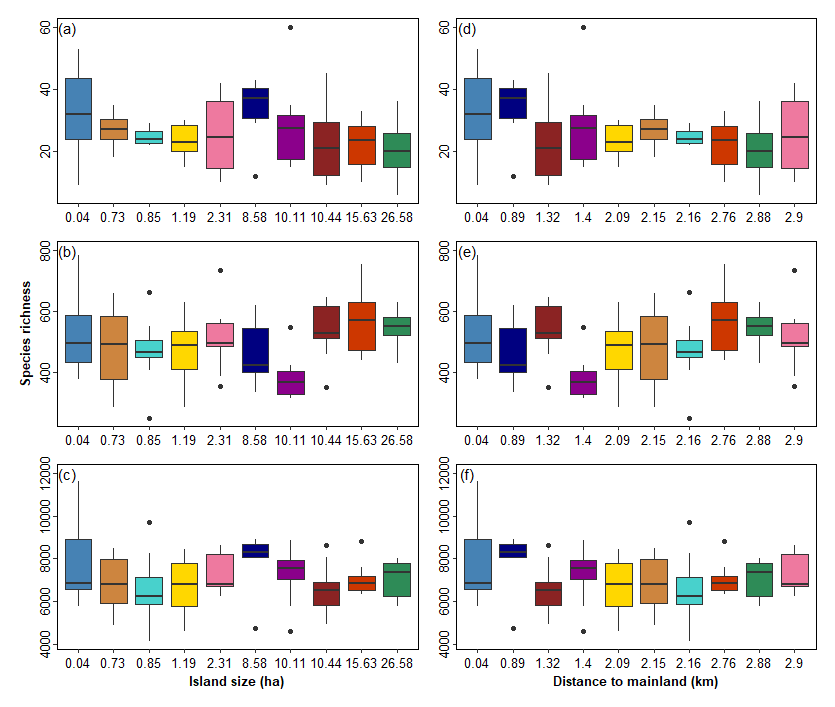
**

**Figure S7**. Boxplots illustrating relationships between island size and species richness of (a) diatoms, (b) cyanobacteria, and (c) non-cyanobacteria and between island distance to mainland and species richness of (d) diatoms, (e) cyanobacteria, and (f) non-cyanobacteria across the rock pools (n = 101). The colors represent different islands. For corresponding island names, see Fig. 3.
