# Supplementary material for: Factors influencing the biodiversity of three microbial groups within and among islands of the Baltic Sea
Source: FEMS Microbiol Ecol. 2021 Mar 22;97(5):fiab049. doi: 10.1093/femsec/fiab049 (PMC8044292; doi:10.1093/femsec/fiab049)
Supplement: fiab049_Supplemental_Files [file fiab049_supplemental_files.zip › FEMS_Manuscript_Revised_SuppInfo_Captions.docx]

**SUPPORTING INFORMATION CAPTIONS**

**Table S1**. Results of redundancy analysis.

**Figure S1**. Spatial autocorrelation among all rock pools.

**Figure S2**. Spatial autocorrelation among freshwater rock pools.

**Figure S3**. Spatial autocorrelation among brackish rock pools.

**Figure S4**. Partial dependence plots of boosted regression tree analyses among all rock pools.

**Figure S5**. Partial dependence plots of boosted regression tree analyses among freshwater rock pools.

**Figure S6**. Partial dependence plots of boosted regression tree analyses among brackish rock pools.

**Figure S7**. Relationships between island size or distance to mainland and microbial species richness.
